# Impact of obstructive sleep apnea on the occurrence of restenosis after elective percutaneous coronary intervention in ischemic heart disease

**DOI:** 10.1186/1465-9921-9-50

**Published:** 2008-06-03

**Authors:** Stephan Steiner, Per O Schueller, Marcus G Hennersdorf, Dominik Behrendt, Bodo E Strauer

**Affiliations:** 1 Department of Cardiology, Pneumology and Angiology University Düsseldorf , 40225 Düsseldorf, Germany

## Abstract

**Rationale:**

There is growing evidence that obstructive sleep apnea is associated with coronary artery disease. However, there are no data on the course of coronary stenosis after percutaneous coronary intervention in patients with obstructive sleep apnea.

**Objectives:**

To determine whether sleep apnea is associated with increased late lumen loss and restenosis after percutaneous coronary intervention.

**Methods:**

78 patients with coronary artery disease who underwent elective percutaneous coronary intervention were divided in 2 groups: 43 patients with an apnea hypopnea – Index < 10/h (group I) and 35 pt. with obstructive sleep apnea and an AHI > 10/h (group II). Late lumen loss, a marker of restenosis, was determined using quantitative coronary angiography after 6.9 ± 3.1 months.

**Main results:**

Angiographic restenosis (>50% luminal diameter), was present in 6 (14%) of group I and in 9 (25%) of group II (p = 0.11). Late lumen loss was significant higher in pt. with an AHI > 10/h (0.7 ± 0.69 mm vs. 0.38 ± 0.37 mm, p = 0.01). Among these 35 patients, 21(60%) used their CPAP devices regularly. There was a marginally lower late lumen loss in treated patients, nevertheless, this difference did not reach statistical significance (0.57 ± 0.47 mm vs. 0.99 ± 0.86 mm, p = 0.08). There was no difference in late lumen loss between treated patients and the group I (p = 0.206).

**Conclusion:**

In summary, patients with OSA and coronary artery disease have a higher degree of late lumen loss, which is a marker of restenosis and vessel remodeling after elective percutaneous intervention.

## Background

Obstructive sleep apnea (OSA) is a common disorder defined by upper airway obstruction, apnea and nocturnal hypoxia. There is a prevalence of OSA in patients with coronary artery disease of up to 50% [1-3]. Beyond this high prevalence, the occurrence of OSA is associated with an advanced state of atherosclerosis [4] and a worse prognosis in these patients [5-7]. In the last decade, there is growing evidence that OSA acts as a cardiovascular risk factor, independent of associated traditional risk factors (e.g. arterial hypertension, dyslipedemia, obesitas).

Percutaneous transluminal coronary angioplasty (PTCA) has proved effective in reducing myocardial ischemia and clinical symptoms in patients with coronary artery disease (CAD) with a primary success rate ranging from 90%–95% in the general population. Although there are promising developments in interventional cardiology, late restenosis is still an unsolved problem of interventional procedures. Hemodynamic restenosis occurs after a period of about 12 Weeks in 30–45% of the cases treated with PTCA [8,9] and 20–30% of the cases with additional coronary stent implantation using bare metal stents [10].

Yumino et al. found a high prevalence of OSA in patients with acute coronary syndrome. In these patients OSA appeared to be an independent predictor of clinical and angiographic outcomes after percutaneous coronary intervention (PCI) [11]. However, there are no data on the course of coronary artery disease after elective PCI in stable patients with OSA. We hypothesized, that OSA is associated with higher occurrence of restenosis after percutaneous coronary intervention.

## Patients and methods

### Patients

Candidates for participation were consecutive patients undergoing elective coronary angiography and percutaneous coronary intervention and clinical suspected nocturnal breathing disorders (heavy snoring, obesity, daytime sleepiness, history of witnessed apneas). All patients underwent overnight-polygraphy (Schwarzer, Germany [12]) between 10.00 p.m and 6.00 a.m. and were classified as sleep apneics or controls according to data of the apnea hypopnea index (AHI). Oronasal airflow was registered using a thermistor, abdominal and thoracic respiration efforts were measured using impendance plethysmography. Oxygen saturation (SaO_2_) was measured using finger pulse oxymetry. The AHI was calculated as the number of respiratory events per hour after manual scoring. Minimal nocturnal oxygen saturation was defined as the lowest saturation reached during sleep after manual exclusion of clear artefacts. As adopted in previous studies [7,12] a threshold AHI of 10/h was accepted as a diagnostic indicator for obstructive sleep apnea syndrome. Cardiovascular risk factors were defined as described in a recent study [12]. The study complied with the declaration of Helsinki. All procedures were carried out as routine procedures, regardless of the study protocol. All patients gave their informed consent.

### Treatment of OSA

All patients with an AHI > 10/h were offered CPAP therapy. Patients with OSA were divided in two groups based on whether they were treated with CPAP. When CPAP was accepted, titration was performed during a second night in the sleep laboratory using an Auto-CPAP device supervised by an experienced doctor (Somnosmart, Weinmann Germany). The P95 read out from the titration device was used to calculate constant CPAP [13]. The treatment group comprised all patients who accepted CPAP therapy, long term compliance was evaluated based on a personnel questionaire. Patients were considered to be CPAP compliant if they used CPAP on an average > 5 h per night, determined at follow up. CPAP therapy was initiated after the PCI and was performed until the date of the second angiographic study.

### Coronary angiography, percutaneuous coronary intervention, quantification of Restenosis

Selective coronary angiography was performed following the administration of intracoronary glyceryl nitrate. At least six standardized projections of the left coronary artery and two of the right coronary artery were obtained. Quantitative analysis of the angiograms was performed (Quantcor, Siemens, [14]) at baseline and at follow up. Before the intervention all patients received 500 mg acetyl salicylic acid (ASA) i.v. and 5000–7500 iE Heparin (activated clotting time (ACT) > 300 sec.). Regular medication includes ASA 100 mg p.o. in all patients and additionally clopidogrel after stent implantation (300 mg loading dose and 75/mg/d over 4 weeks). Coronary stents (bare metal stents) were implantated in case of coronary dissection or elastic recoil, as well as in calcified stenoses with deficient results of balloon-angioplasty alone.

### Follow up

Follow up coronary angiography was carried out in every patient as a routine procedure after 6.9 ± 3.1 months, regardless of the presence of clinical symptoms or results from non-invasive measurements of myocardial ischemia. Clinical relevant restenosis was defined as > 50% stenosis of the initial target lesion at follow up. Late luminal loss was determined using quantitative coronary arteriography (minimum luminal diameter immediately after angioplasty minus minimal luminal diameter at follow up).

### Exclusion criteria

Exclusion criteria were: acute coronary syndrome, use of drug eluting stents, failed angioplasty with a more than 50% residual stenosis and a reduced TIMI flow after the PCI.

### Statistics

The data were analyzed with the Statistical Package for Social Sciences (SPSS 11.0 for Windows, Munich Germany). For comparison of several groups the Mann-Whitney U Test was used. Non-continuous data were analyzed using the two tailed Fisher exact test. Correlation coefficients were generated with the Spearman test. A multivariate logistic regression analysis was performed to assess the predictive variables of late lumen loss. The included variables were selected, if they were significant during univariate analysis or were considered to be biologically relevant. Significant difference between groups was assumed at the level of error < 5%. Tests between 5% and 10% were considered as statistical trends.

## Results

Between 2001 and 2005 78 patients were included in the study. Analysis of quantitative angiographic variables showed, that the severity of the coronary stenosis (per cent diameter stenosis before the intervention (r = -0.385, p = 0.001) and immediately after the procedure (r = 0.674, p = 0.001)) was positively correlated with late lumen loss, indicating, that severity of vessel injury is a promotor of restenosis. There was no significant correlation between late lumen loss and maximal balloon pressure (r = -0.077, p = 0.522), or vessel diameter (r = 0.053, p = 0.66).

Clinical characteristics were similar in patients with or without sleep apnea, in both groups most of the patients were men (see Table 1). There was a high prevalence of cardiovascular risk factors. The proportion of patients with a positive smoking history, arterial hypertension, hyperlipoproteinemia, obesity or diabetes mellitus were similar in both groups, as was the number of risk factors per patient (3.19 ± 1.03 vs. 3.05 ± 1.05) (see Table 2). There was no difference in left ventricular systolic function (Ejection fraction (66 ± 12% vs. 64 ± 16%)).

**Table 1:** Clinical characteristics and cardiovascular treatment at baseline. Plus/minus values are means ± SD.

	**Control n = 43**	**OSA n = 35**	**p**
Age (y)	65.1 ± 9.6	67.4 ± 7.3	0.17
Male n (%)	39 (90.7)	32 (91.4)	0.66
BMI (kg/m^2^)	27.9 ± 4.6	28.1 ± 4.0	0.82
AHI (events/h)	3.8 ± 2.5	27.5 ± 16.6	< 0.001
Min SaO_2 _(%)	89.3 ± 3.2	83.8 ± 4.0	< 0.001
Medication			
Platelet inhibitor, n (%)	40 (93)	33 (94.3)	0.82
Beta Blocker, n (%)	33 (76)	25 (71.4)	0.59
ACE Inhibitor, n (%)	30 (69.8)	25 (71.4)	0.87
Statin, n (%)	34 (79.1)	26 (74.3)	0.62

**Table 2:** Cardiovascular risk profile in control and obstructive sleep apnea

	**Control n = 43**	**OSA n = 35**	**p**
HTN n (%)	35 (81.3)	27 (77)	0.64
RR_sys _(mmHg)	133 ± 17	135 ± 15	0.51
RR_dia _(mmHg)	78 ± 11	77 ± 10	0.64
HLP, n (%)	40 (93)	34 (97)	0.82
Smoker/Ex-Smoker, n (%)	3 (6.9)/22 (51.1)	5 (14.2)/21 (60)	
Packyears (n)	15 ± 19	22 ± 22	0.16
Diabetes mellitus, n (%)	8 (18.6)	8 (22.8)	0.64
Family History (CAD), n (%)	17 (39.5)	15 (42.8)	0.96
Number of Risk factors per patient	3.19 ± 1.03	3.05 ± 1.05	0.73
Laboratory variables			
Total Cholesterol (mg/dl)	193 ± 41	190 ± 43	0.63
LDL Cholesterol (mg/dl)	126 ± 38	120 ± 31	0.40
HDL Cholesterol (mg/dl)	52 ± 20	49 ± 12	0.51
LDL/HDL	2.8 ± 1.3	3.1 ± 4.0	0.66

The predominant target vessel for intervention was the left anterior descending artery (LAD) in both groups, and there were no significant differences in complexity and angulation of stenoses that were dilated.

Stent implantation was performed in 22 (51%) patients with an AHI < 10/h, and in 23 (64%) patients of group II (n.s.). There were no significant differences in respect of periprocedural variables, such as balloon size or inflation time between the two groups (see Table 3). Angiographic restenosis, defined by the presence of a hemodynamically relevant stenosis (>50% luminal diameter), was present in 6 (14%) of group I and in 9 (25%) of group II (p = 0.11). Late lumen loss was significantly higher in OSA-patients (0.7 ± 0.69 mm vs. 0.38 ± 0.38 mm, p = 0.01).

**Table 3:** Angiographic findings and periprocedural variables in patients with and without obstructive sleep apnea.

	**Control n = 43**	**OSA n = 35**	**p**
Follow up period (months)	7.1 ± 2.7	6.7 ± 3.7	0.43
Ejection fraction (%)	66 ± 12	64 ± 16	0.84
Coronary artery narrowed (RCA/LAD/LCX)	14/20/9	8/17/10	
Type of lesion A/B/C	20/17/6	17/11/7	
Reference diameter (mm)	2.89 ± 0.43	3.04 ± 0.56	0.18
Final balloon size (mm)	2.98 ± 0.42	3.01 ± 0.47	0.76
Maximal inflation pressure (atm)	11.2 ± 2.8	10.4 ± 2.5	0.20
Total inflation time (sec)	89 ± 53	78 ± 45	0.38
Stent, n (%)	22 (51)	23 (64)	0.11
Restenosis > 50%, n (%)	6 (14)	9 (26)	0.11
Late lumen loss (mm)	0.38 ± 0.38	0.7 ± 0.69	0.01

Stepwise multiple linear regression analyses were conducted to determine relations of gender, age, BMI, cardiovascular risk factors (diabetes mellitus, arterial hypertension, hyperlipoproteinemia) and lesion morphology with late lumen loss. An apnea hypopnea index > 10/h, and minimal luminal diameter of the coronary segment were significant predictors of late lumen loss. An AHI > 10/h remained a significant predictor of late lumen loss after adjusting for cardiovascular risk factors as diabetes mellitus, hypertension, hyperlipidemia and body mass index.

**Figure 1:**
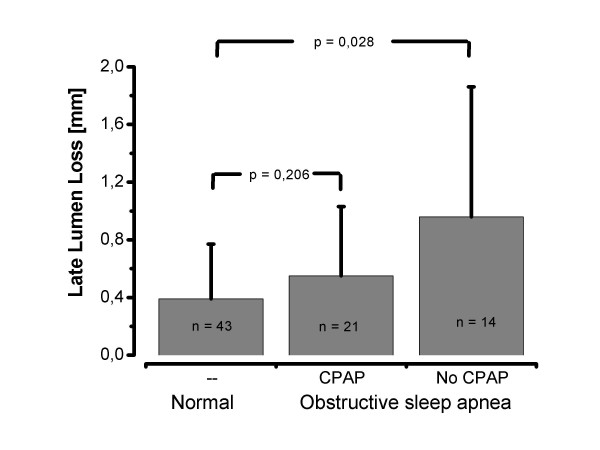
Late lumen loss in patients without obstructive sleep apnea, OSA patients without treatment and OSA patients with effective CPAP therapy.

Among the 35 patients with an AHI > 10/h, 21 (60%) accepted treatment with CPAP and used their devices regularly. Although CPAP users had a higher BMI, there was no difference in apnea hypopnea index or minimal nocturnal oxygen saturation at baseline. There was a marginally lower late lumen loss in treated compared to non-treated OSA patients, nevertheless, this difference did not reach statistical significance (0.57 ± 0.47 mm vs. 0.99 ± 0.86 mm, p = 0.08) (see Table 4). There was no significant difference in late lumen loss after percutaneous coronary intervention between group I and treated patients of group II (Fig. 1).

**Table 4:** Angiographic findings and periprocedural variables in patients with obstructive sleep apnea with regard to CPAP treatment.

	**No CPAP n = 14**	**CPAP n = 21**	**p**
Severity of OSA			
BMI (kg/m^2^)	26.4 ± 3.3	29.3 ± 4.2	0.04
AHI (ev/h)	26.3 ± 17.6	27.4 ± 16.0	0.8
Min SaO_2 _(%)	83.6 ± 4.3	82.8 ± 3.9	0.9
Arteriogramm and lesion characteristics			
Coronary artery narrowed (RCA/LAD/LCX)	2/9/3	6/8/7	
Type of lesion A/B/C	9/2/3	8/9/4	
Reference diameter (mm)	3.0 ± 0.6	3.1 ± 0.6	0.82
Final balloon size (mm)	3.1 ± 0.5	2.9 ± 0.4	0.25
Maximal inflation pressure (atm)	9.4 ± 1.8	11 ± 2.8	0.07
Total inflation time (sec)	70 ± 36	84 ± 50	0.41
Stent, n (%)	7 (50)	15 (71)	0.09
Restenosis > 50%, n (%)	4 (28.6)	5 (23.8)	0.32
Late loss (mm)	0.99 ± 0.86	0.57 ± 0.47	0.08
Ejection fraction (%)	61 ± 15	67 ± 16	0.28

## Discussion

Although there is growing evidence that obstructive sleep apnea is associated with coronary artery disease and cardiovascular events, this is the first study which focuses on the problem of restenosis after elective coronary intervention in these patients. Based on quantitative coronary angiography, late lumen loss, which is a marker of restenosis and vascular remodeling, was enhanced in OSA-patients. The rate of hemodynamically relevant angiographic restenosis >50% was almost 2-fold higher in patients with OSA (25%) compared to patients without sleep apnea (14%), although there was no statistically significance. Sleep apneics who regularly performed CPAP showed a slight decrease of late lumen loss, implicating, that this therapy might have beneficial effects with regard to restenosis and the clinical course of coronary artery disease in OSA patients.

There are several pathomechanisms contributing to cardiovascular risk in OSA: increase of sympathetic nervous system activity [15], decrease in heart rate variability, endothelial damage and dysfunction [16,17], platelet activation, increase in blood coagulability [12] and insulin resistance [18]. In a 7 year follow up study, patients with OSA had a 4.9 fold greater risk of developing cardiovascular disease during the follow up, independent of other risk factors [1] Our data support the hypothesis, that coronary occlusion might be one reason for the worse prognosis and outcome in these patients. Further on, the data implicate, that OSA-patients carry an increased risk of restenosis potentially associated with clinical events following percutaneous coronary interventions. This is in concert with a recent study which found OSA to be associated with increased cardiac death after percutaneous coronary intervention [19].

Follow up studies in patients undergoing balloon angioplasty showed renarrowing at the side of angioplasty to be a gradual, time-related phenomenon which appeared to reach a zenith at 4–6 month [8,20]. There are different aspects of the late result of coronary intervention: the outcome of the patients, and, from the anatomical point of view, the angiographic result determined by the diameter of the vessel/lesion site at its narrowest point ("minimal luminal diameter"). The renarrowing occurring from immediately after the intervention over the following 6 months as determined and quantified by the follow angiogramm conveys the degree of new tissue growth and vessel remodeling [8], factors, which might be influenced by intermittend nocturnal hypoxemia in patients with OSA. In this regard, there is only one study investigating the contribution of nocturnal hypoxemia to the development of restenosis after percutaneous coronary intervention. Hayashi et al. [21] used nocturnal oxymetry as a screening tool for OSA after stent placement in a small group of 35 patients with coronary artery disease. They suggested, that nocturnal hypoxemia may be associated with coronary restenosis. Nevertheless, confirmation of the diagnosis of sleep apnea syndrome could not be established. Milleron et al. [5] report on a group of 54 patients with sleep apnea and coronary artery disease. They found, in concert with our findings, that OSA was associated with a higher rate of cardiovascular events e.g. revascularization or myocardial infarction in untreated OSA patients.

There is evidence, that restenosis is affected by inflammatory processes [22]. It is supposed, that nocturnal hypoxemia causes inflammation. In this regard it was shown, that OSA is associated with an elevated C-reactive proteine [23,24], Interleukin-6 [24], serum amyloid A [25] and elevated Fibrinogen and plasma viscosity [12]. In addition, most of these parameters were normalized using CPAP-Therapy in patients with OSA, indicating a causative role of OSA in the inflammatory process. Since inflammation might play a central role in renarrowing of the vessels in OSA patients, the role of drug eluting stents has to be assessed in these patients.

CPAP therapy is recommended in any OSA patient with an AHI exceeding 30/h or at a minimal threshold of 5/h if the patient is suffering symptoms like daytime sleepiness, impaired cognition, insomnia or cardiovascular disease [26]. Futhermore, recent studies support a protective effect of CPAP therapy with regard to death from cardiovascular disease in patients with OSA [27] and indicate, that CPAP is associated with a decrease in the occurrence of new cardiovascular events, and an increase in the time to such events [5]. According to the results of the Sleep Heart Health Study [28] it seems prudent to advocate CPAP therapy in patients with CAD and moderate OSA, even if they do not suffer from excessive daytime sleepiness [8]. In our study, only 60% of the OSA patients accepted CPAP and used their device regularly. However, this rate might be optimized if CPAP is not only recommended as a means of controlling symptoms of OSA but also as part of their CAD treatment. In this regard it was shown, that adherence to CPAP might reach nearly 100% in patients with coronary artery disease and sleep apnea, even without daytime sleepiness [29]. By all means, patients with risk profile for OSA (e.g. obesity, sleepiness) should be screened for nocturnal breathing disorders to optimize cardiovascular risk and the risk of restenosis after percutaneous coronary intervention.

### Limitations of the study

There are several limitations of the study: first of all, we did not carry out overnight polysomnography, therefore we can not rule out sleep relating breathing disorders in all patients in group I. Still, minimal oxygen saturation and AHI are the common parameters describing the severity of nocturnal breathing disorders. Furthermore, there were no follow up sleep studies at the time of the second angiography study. Another limitation refers to the study design, since there was no randomization of the OSA patients with regard to CPAP. Therefore, we can not exclude some misclassification bias. In this regard, there was a higher rate of stent placements in patients with CPAP-therapy compared to patients without CPAP-therapy within the OSA group, which might have contributed to the less pronounced late lumen loss in CPAP treated patients. Further limitation refers to the study design, which does not allow to verify a causal relationship.

In summary, patients with OSA and coronary artery disease have a higher degree of late lumen loss, which is a marker of restenosis and vessel remodeling after elective percutaneous intervention.

## Abbreviations

LAD: Left anterior descending artery; LCX: Left circumflex coronary artery; RCA: Right coronary artery; PCI: Percutaneous coronary intervention; PTCA: Percutaneous transluminal coronary angioplasty; AHI: Apnea hypopnea index; CPAP: Continuous positive airway pressure; OSA: Obstructive sleep apnea; ACT: Acitvated clotting time; BMI: Body mass index; CAD: Coronary artery disease; EF: Left ventricular ejection fraction.

## Authors' contributions

SS conceived for the study, carried out the sleep studies and drafted the manuscript

PS participated in the sequence alignement, acquisition of data and follow up

MH carried out coronary angiography and quantitative coronary angiography

DB carried out quantitative coronary angiography and performed statistical analysis

BS participated in the study design, interpretation of results and study coordination

All authors read and approved the final manuscript.
